# Interfacial Insight of Charge Transport in BaTiO_3_/Epoxy Composites

**DOI:** 10.3390/nano13030406

**Published:** 2023-01-19

**Authors:** Beibei Jia, Jun Zhou, Jiaxin Chen, Zixuan Zhang, Yang Wang, Zepeng Lv, Kai Wu

**Affiliations:** 1 Center of Nanomaterials for Renewable Energy, State Key Laboratory of Electrical Insulation and Power Equipment, Xi’an Jiaotong University, Xi’an 710049, China; 2 School of Electronics and Information, Xi’an Polytechnic University, Xi’an 710048, China

**Keywords:** interface, epoxy resin, charge transport, trap

## Abstract

Space charge accumulation greatly influences the dielectric performance of epoxy composites under high voltage. It has been reported that nano-fillers can suppress the charge accumulation in the bulk of insulation materials. However, it is still unclear how the nano-fillers influence the charge distribution at the interface between the filler and polymeric matrix. In this work, the dielectric properties and the local dynamic charge mobility behavior at the interface of barium titanate/epoxy resin (BTO/EP) composites were investigated from both bulk and local perspectives based on the macroscopic test techniques and in-situ Kelvin probe force microscopy (KPFM) methods. Charge injection and dissipation behavior exhibited significant discrepancies at different interfaces. The interface between BTO and epoxy is easy to accumulates a negative charge, and nanoscale BTO (n-BTO) particles introduces deeper traps than microscale BTO (m-BTO) to inhibit charge migration. Under the same bias condition, the carriers are more likely to accumulate near the n-BTO than the m-BTO particles. The charge dissipation rate at the interface region in m-BTO/EP is about one order of magnitude higher than that of n-BTO/EP. This work offers experimental support for understanding the mechanism of charge transport in dielectric composites.

## 1. Introduction

In high/ultrahigh voltage (HV/UHV) direct current (DC) power transmission systems, the insulating performance of dielectric materials is vital for the safety of electrical equipment [1,2,3,4]. There will be space charge accumulation inside the insulating material under a high-voltage DC electric field, resulting in the distortion of the electric field [5]. The space charge accumulation accelerates the aging of dielectric materials and causes partial discharge, which will even lead to electric breakdown [6]. These phenomena enhance the risk of failure of insulating materials and shorten the service life of the electrical equipment [7,8,9]. Epoxy resin (EP) composite is widely used in HV/UHV DC transmission equipment. It has excellent physical and chemical properties, such as adhesion, mechanical, electrical insulation, chemical stability, low shrinkage, and easy molding and processing [10,11,12]. However, pure EP has high cross-linking density after curing, so it often suffers from disadvantages such as large internal stress, brittleness, and poor heat stability, which essentially limits its application [13,14,15]. Therefore, the improvement of the dielectric property of the material has attracted great attention for researchers by introducing inorganic fillers into epoxy in recent years [16,17].

It has been found that the thermal and electrical properties of dielectric materials can be significantly improved by adding micro or nano-scale boron nitride, alumina, carbon nanotubes, and other inorganic or organic fillers [18,19,20,21,22]. These embedded fillers with larger specific surface areas will inevitably introduce a large number of interface areas into the epoxy matrix [23,24]. Additionally, the interface plays a critical role in determining the dielectric properties of composites [25,26,27]. At the same time, there are lots of interface traps that can capture and hinder the movement of carriers in the interface region [28,29,30]. Wang et al. reported that interface has obvious effects on through-plane thermal conductivity and dielectric properties by intercalating the hybrid fillers of the alumina and boron nitride nanosheets (BNNS) into epoxy resin [31]. Peng et al. discovered that sLTNO@mAC/EP composites synthesized by grafting Li_0.3_Ti_0.02_Ni_0.68_O particles (sLTNO) on the surface of carbon nanotube bundles (mAC) and before being embedded in EP have a higher dielectric constant and lower dielectric loss, which mainly depend on the interface polarization between the filler and matrix [32]. Peng et al., found that there is indeed a phenomenon of local interface polarization between copolymer vinylidene fluoride and trifluoroethylene (P(VDF-TrFE)) and nanoparticles [33]. 

However, at present, it is still unclear how the fillers affect the charge accumulation or dissipation in the dielectric composite materials [34,35,36]. Even though some studies have discussed the polarization and charge distribution at the interface region in dielectric composites using classical interface models, such as Tanaka’s multi-core model and Lewis’s electrical double layer model et al. [37,38,39,40], it is hard to directly elucidate and monitor the charge transport behavior at the micro- or nano-scale using experiment tests. KPFM technology could directly obtain the surface potential at the local region of dielectrics, thus providing a powerful method to investigate the mechanism of charge distribution on a microscopic level. For instance, Devon et al. indicated that PF-KPFM could be used to reveal temporal dynamics of surface potential [41]. Faliya et al. reported that the complexity of the space charge formation and movement inside poly(ethylene oxide)-based electrolytes had been studied via KPFM technology [42]. Peng et al. found that the size of the interface is larger than that of particles in low density polyethylene/Titanium oxide (LDPE/TiO_2_) nanocomposite by electrostatic force microscope (ImEFM) technology [43]. Although previous works have studied charge migration behavior in dielectric materials, there is a lack of research on the charge distribution and mobility at the interface region between the filler and polymeric matrix.

This paper furthers research into the influence of filler on charge distribution and mobility characteristics on a micro-/nanometer scale in the dielectric composites based on our previous study [44] so as to deeply clarify the micro mechanism whereby the introduction of fillers improves the macroscopic dielectric properties of materials. Different from the existing reports, which applied vertical bias through the tip of KPFM, in this work, the external voltages were applied in a horizontal direction on the BTO/EP composite, which can effectively inhibit the capacitance effect between the tip and selected area of the sample. Two metal-Al electrodes were deposited on the BTO/EP composite films by vacuum-heat-evaporation technology with the help of a metal mask. High electrical field strength was achieved by reducing the gap between two electrodes in the horizontal direction, as shown in Figure S1. Concurrently, the pulsed-electro-acoustic (PEA) method was used to elucidate the dynamic of space charge in BTO/EP bulk composites at the macroscopic level, and the dielectric properties were also studied. Moreover, the interfacial mechanism was also analyzed by combining the macroscopic and microscopic experimental results. Our work provides significant insight into understanding local charge transport behavior at the interfacial region in insulating composites. 

## 2. Materials and Methods

### 2.1. Raw Materials

Epoxy resin (E51,WSR618) was purchased from Nantong Xingchen Synthetic Materials Co., Ltd. (Nantong, China) Curing agent (Methy Tetrahydrophthalic Anhydride) was obtained from Nantong Runxiang New Material Co., Ltd. (Nantong, China) Accelerator (N,N-Dimethylbenzylamine, CP) was obtained from Sinopharm Chemical Reagent Co., Ltd. BTO powers were purchased from Shanghai Aladdin Biochemical Technology Co., Ltd. (Shanghai, China) In this work, two different sizes of BTO particles were used to be fillers and their sizes are 1 μm and 450 nm, respectively, and the different sizes of particles are later labeled as m-BTO and n-BTO, separately. 

### 2.2. Sample Preparation

**Figure 1:**
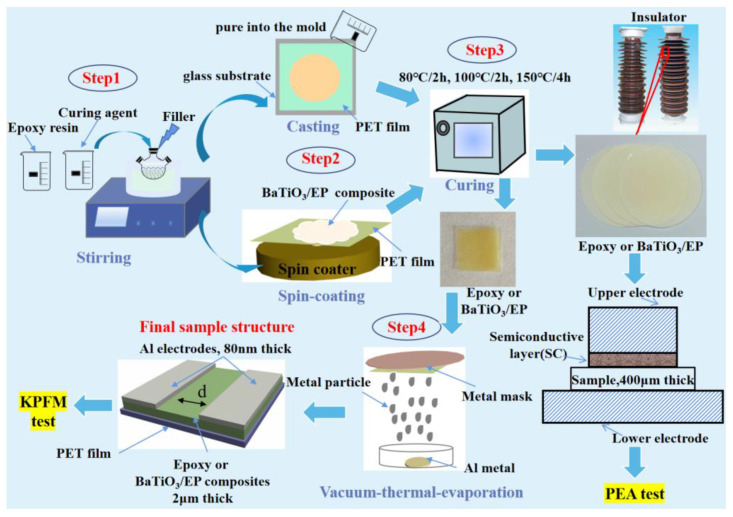
Schematic diagram of the fabrication of BTO/EP composites.

Fabrication of the BTO/EP composites with a different structure for the KPFM and macro tests has been schematically depicted in Figure 1. A thin film of BTO/EP composite with a thickness of approximately 2 μm was prepared from its solution on a Polyester (PET) film using a spin coating technique. Initially, the BTO powders were dried at 60 °C for 12 h in a vacuum oven and then mechanically premixed for 15 min with the curing agent at a speed of 2400 rpm at room temperature. Subsequently, the mixture was ultrasonically treated for 30 min in the ultrasonic cleaner. After that, the mixture was poured into a three-necked flask containing epoxy resin which was degasified at 60 °C for 30 min under a vacuum and stirred by a magnetic stirrer at 60 °C for 30 min in the vacuum condition. The epoxy resin, curing agent, and accelerator were mixed with a weight ratio of 100:80:0.3. For the KPFM test, the mixture liquid was spin-coated on a PET polyester film with the thickness of ~0.08 mm by the vacuum spin coater at a speed of 800 rpm for 30 s and then 1000 rpm for 30 s, finally followed by curing processing. The samples used for the macroscopic test were prepared as follows: the above mixed liquid was poured into the molds for casting and then cured in a vacuum oven at 80 °C for 2 h, 100 °C for 2 h, and 150 °C for 5 h, achieving BTO/EP composites with the thickness of around 0.15 mm or 0.38 mm after demolding processing. A pair of metal-Al electrodes were evaporated on the surface of the sample. The thickness of the electrodes was set to 80 nm in the electrode preparation process. The actual thickness of the two electrodes is shown in Figure S2a. The distance *d* between the two electrodes is about 35 μm, as shown in Figure S2b.

### 2.3. Characterization

Field emission scanning electron microscopy (SEM, GeminiSEM 500, Carl Zeiss (Shanghai, China) Co., Ltd.) was employed to reveal the fractured surface morphologies of the BTO/EP composites. The fractured surfaces of the samples were sprayed with a thin gold layer before observation. The composites were immersed in liquid nitrogen and then quickly fractured. The structure analysis of the BTO particle was characterized by an X-ray diffractometer (XRD, D8 Advance, Bruker, Ltd., Nasdaq, USA.). The glass transition temperature was obtained using differential scanning calorimetry (DSC, DISCOVER DSC250, TA instrument). The dielectric properties of the composites were analyzed by a broadband dielectric spectrometer (concept 80, TE) in the frequency range from 0.1 Hz to 1 MHz. A PEA test platform system was used to measure the bulk space charge distribution. The conductivity of the composites at different field strengths was tested by 6517B (a self-built high-voltage DC conductivity test platform system). Breakdown field strength was collected by a high-voltage DC breakdown test system.

### 2.4. KPFM Measurement

**Figure 2:**
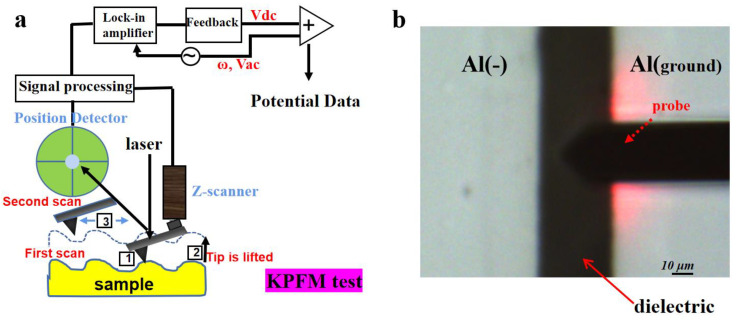
Schematic diagram for KPFM test (**a**); Photo of the local area in the test sample taken by optical microscope (**b**).

The KPFM test is performed in lift mode along a line between two electrodes to obtain the surface potential data, as shown in Figure 2a. In the first scan, the height profile of the sample was recorded. In the second scan, the cantilever was lifted up to 90 nm, and scanned the surface potential by following the particular line which previously measured the height profile. The scanning rate was 0.1 Hz. In this frequency, it gives enough time to the feedback system to respond appropriately to the changes in height and surface potential so that a better resolution with the minimum noise level can be obtained [45]. Additionally, a sufficiently flat surface was required to improve lateral resolution. Platinum–iridium-coated conductive probes (SCM-PIT, Bruker) were used. Figure 2b shows the local topography of the test sample using an optical microscope. The environmental humidity is controlled to be about 1ppm to avoid the effect of the atmospheric water. All KPFM measurements were carried out in the glove box filled with N_2_ at room temperature; the experimental setup is shown in Figure S3.

## 3. Results and Discussion

### 3.1. Morphology of the BTO/EP Composite

**Figure 3:**
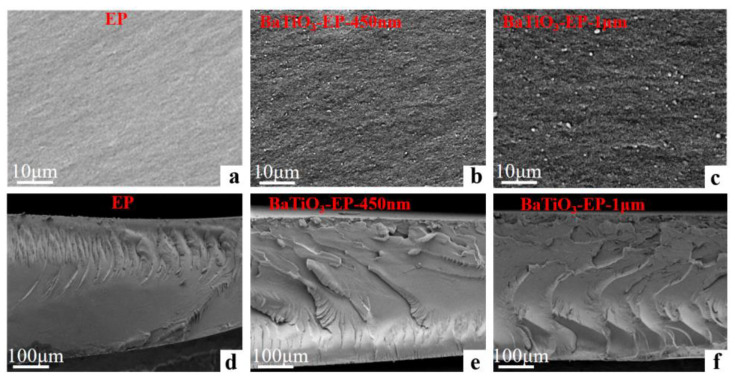
SEM images of the fracture surfaces for pure epoxy resin (**a**,**d**), n-BTO/EP (**b**,**e**), and m-BTO/EP composites (**c**,**f**).

The optical morphologies images of pure epoxy and 1.0 wt% BTO/EP composites are shown in Figure 3. The BTO fillers were uniformly dispersed in the epoxy matrix (Figure 3a–c), and there are obviously discontinuous states between the matrix and BTO particles. In other words, the interfaces of both n-BTO and EP, as well as m-BTO and EP, are clearly visible in the BTO/EP composites. In addition, the low-magnification images reveal the information in detail for the propagation direction of the cracks in different materials, from left (right) to right (left) in a horizontal direction, as depicted in Figure 3d–f, The m-BTO/EP composites exhibit greater toughness compared with pure epoxy resin and n-BTO/EP composite. Furthermore, the addition of n-BTO particles significantly enhanced the thermal stability of the materials, as shown in Figure S4.

### 3.2. Dielectric Properties of the BTO/EP Composite

**Figure 4:**
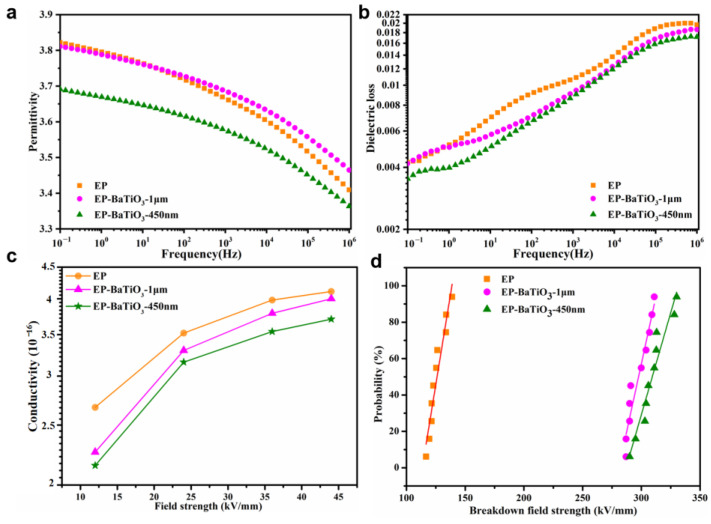
Variation of permittivity (**a**) and dielectric loss (**b**) as a function of the logarithm of frequency for the unfilled EP and BTO/EP composites; Conductivity as a function of field strength for epoxy composites (**c**); The Weibull statistical distribution of breakdown field strength for neat EP and BTO/EP composites (**d**).

Figure 4a presents the variation of the dielectric constant of EP and BTO/EP composites with a thickness of 0.15 mm versus frequency at room temperature, ranging from 10^−1^ Hz to 10^6^ Hz. The dielectric constant of neat epoxy and its composites with low content (1.0 wt%) decreases with the increase in frequency. This phenomenon is due to the fact that the reorientation of the dipole of the polymer molecular cannot keep up with the change in frequency. The addition of BTO particles delivered a decrease in permittivity with respect to that of pure epoxy, especially for the n-BTO particle, which is ascribed to the fact that the filler particle has an inhibitory effect on the polarization mechanism in the dielectric composites. In comparison with the m-BTO/EP composite, there are larger volume fractions at the interface region in n-BTO/EP material. According to the double nano-meter layer model proposed by Tsagarapoulos et al. [46], the innermost layer is bound to the particle surface, which limits the molecular chain motion and the steering polarization of the dipole, leading to the reduction of the dielectric constant.

The dielectric loss of epoxy composites as a function of frequency is shown in Figure 4b. The dielectric loss of pure epoxy and n-BTO/EP composites increases with frequency, and n-BTO/EP composite has relatively lower dielectric loss than that of m-BTO/EP material. In low-frequency region, the dielectric loss increases with frequency due to the relaxation loss of interfacial polarization and the conductivity loss caused by impure ions. In high-frequency region, the dipole polarization cannot keep up with the change of frequency, resulting in an increase in dielectric loss with frequency.

Figure 4c gives the relationship between the conductivity of single-layer samples with a thickness of ~0.15 mm measured by a three-electrode system and an electric field (10~45 kV/mm) for epoxy composites. In this test, the current values for different samples after polarization of 40 min were taken as the steady-state current, and different electric fields were achieved by continuously increasing the voltage. It is worth mentioning that the conductivity of the BTO/EP composites is always lower than that of pure epoxy under the same conditions, which demonstrates that the addition of BTO particles may introduce a large number of defects into the composite system. These defects capture carriers and limit charge migration. Additionally, the conductivity of the n-BTO/EP composite is lower than that of the m-BTO/EP material. This result implied that the n-BTO particles introduce relativity deeper interface traps into the system so that it has a stronger ability to capture carriers. 

Figure 4d exhibited the Weibull statistical distribution of breakdown field strength for pure EP and BTO/EP composites. In the DC high voltage breakdown test, ball-ball electrodes were used and immersed in insulating oil during the test. Each sample with a thickness of about 0.15 mm was tested ten times as a set of data, and the voltage was increased at a rate of 2 kV/s. The breakdown field strength of the BTO/EP composites displays a significant increase from ~100 kV/mm (pure epoxy) to ~300 kV/mm. More remarkably, the n-BTO/EP composite has higher breakdown strength than that of m-BTO/EP composites. It is speculated that there is a higher positive or negative charge density around n-BTO particles compared with m-BTO particles. The carriers move and accumulate around the BTO particles under Coulomb force, forming a Debye shield layer. When electrons enter the Debye shield layer around the filler, they will lose part of their energy due to scattering or attraction, thus increasing the breakdown field strength of composites.

The above experimental results show that the interface plays a decisive role in the dielectric properties of composite materials.

### 3.3. Space Charge Analysis via the PEA Method

**Figure 5:**
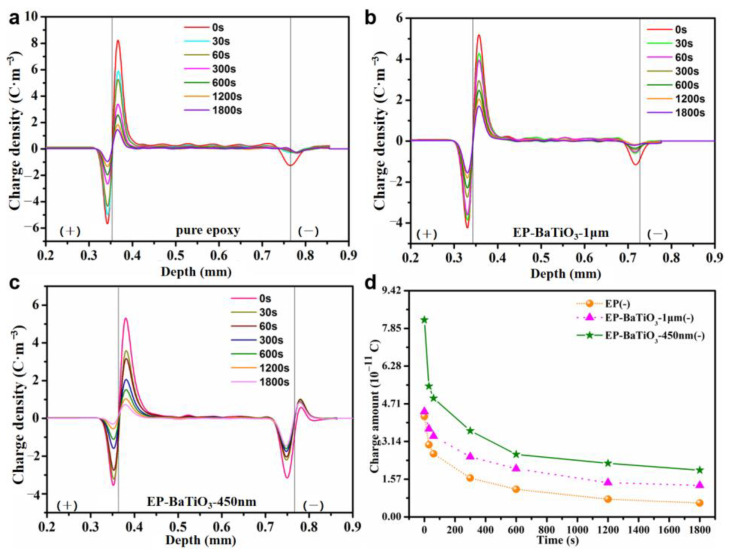
Space charge distribution of pure epoxy (**a**), m-BTO/EP (**b**), and n-BTO/EP (**c**) at different times after removal of voltages; Decay curves of charge amount for pure epoxy and BTO/EP composite during depolarization process (**d**).

The dynamic decay behavior of the space charge in bulk BTO/EP composites was measured by the PEA method after polarization of 40 min at the field strength of 50 kV/mm. Here, the moment of ten seconds after the removal of voltage is chosen as t = 0 s. At this time, the capacitive charge on the electrodes has been completely dissipated. The thickness of the test samples was 0.38 mm. The space charge distribution of pure epoxy and BTO/EP composites are shown in Figure 5a–c. There is homo-polar charge accumulation near the anode in both pure epoxy and m-BTO/EP dielectric materials, while the homo-polar charge gathers near the anode and cathode electrodes in the n-BTO/EP composite. In order to further confirm the charge decay behavior in the dielectric materials, the charge density is integrated along the thickness direction to calculate the average volume density of space charge, and the calculation formula is as follows:

(1) $$q\left(t\right)=\frac{1}{d}\int_{0}^{d}\left|\rho \left(x,t\right)\right|dx$$

where *p*(*x,t*) is the space charge density; *d* is the thickness of test sample. 

We can clearly see that the charge amount Q of composites with n-BTO particles near the anode is much larger than that of m-BTO/EP composite and neat EP, following the sequence: Q_n-BTO/EP_ > Q_m-BTO/EP_ > Q_EP_ at t = 0 s, as plotted in Figure 5d. The remaining charge amount of n-BTO/EP and m-BTO/EP composite materials after depolarization of 1800 s is 2.20 × 10^−11^ C and 1.57 × 10^−11^ C, respectively, and at this time, the decay rate of charge amount with time (i.e., the slope of the curve) for the three samples is almost same. Therefore, in contrast to neat EP and m-BTO/EP composite, the charge dissipation in n-BTO/EP material will take a longer time to complete. Based on the above results, it is supposed that n-BTO particles most likely introduced a deeper trap into the interface region, and the de-trapping of carriers requires higher energy, resulting in the slower charge dissipation rate of n-BTO/EP than that of m-BTO/EP composite.

### 3.4. Charge Distribution and Transport Behavior at the Interface Region of BTO/EP under Bias

**Figure 6:**
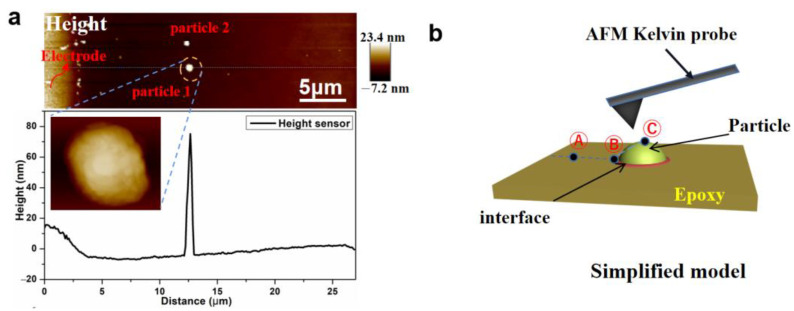
Height diagram of BTO/EP composite with BTO particles (**a**), and Simplified structure configuration of test sample for KPFM test (**b**).

The charge distribution around BTO particles was further investigated by KPFM technology under bias conditions. In order to eliminate the influence of the capacitance effect between tip and metal electrodes, the particles with a distance of more than 10 μm far from the left electrodes were selected. The BTO particle 1 and 2, with a diameter of about 1 μm and 450 nm, respectively, have similar height differences with the epoxy matrix, as shown in Figure 6a and Figure S5b. The XRD spectra of the two types of particles in Figure S5b show that there is typical diffraction (002) peak at around 45°, indicating that BTO powders have tetragonal crystal structure [47]. To further elucidate the charge mobility behavior around the two BTO particles, Figure 6b exhibits three locations that separately stand for epoxy matrix (Ⓐ), interface (Ⓑ), and BTO particle (Ⓒ). 

**Figure 7:**
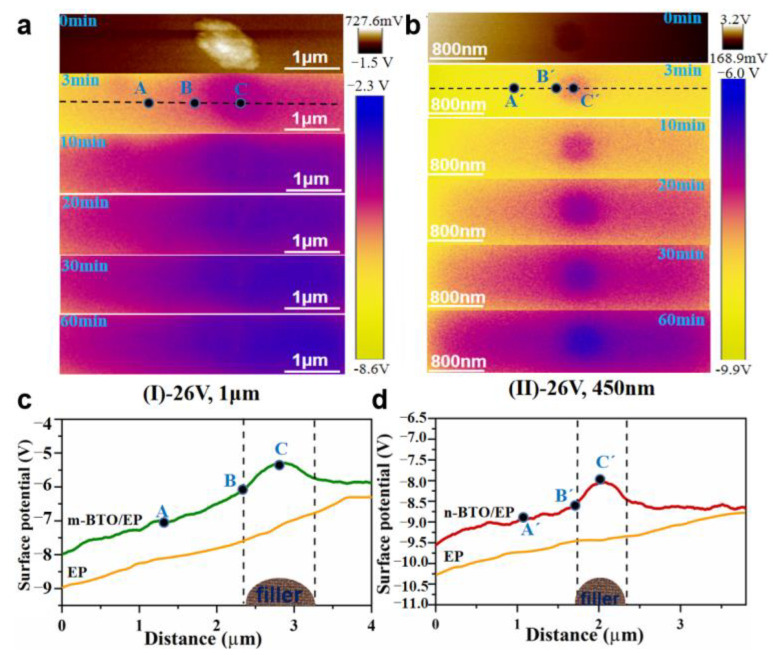
Surface potential images of m-BTO/EP (**a**) and n-BTO/EP (**b**) under −26 V bias at different times; Surface potential curves of m-BTO/EP (**c**) and n-BTO/EP (**d**) obtained around a single BTO particle.

To visualize the charge injection process around a single m-BTO and n-BTO particle embedded in matrix, potential images were used to record the time response under −26 V bias, as shown in Figure 7a,b. It is worth mentioning that the left electrode is charge injection side, and the right electrode is grounded. We can easily observe the discrepancy in color between the BTO particle and matrix at different times, and the shade of the color represents the magnitude of surface potential value. The obvious discrepancy in surface potential between the two phases before applying the external voltage can be assigned to the difference in permittivity between the BTO particle and EP matrix (Figure S5a), leading to interfacial polarization [48]. Figure 7c,d indicated that the surface potential around the two BTO fillers fluctuates greatly compared with that of the pure epoxy, and the surface potential value at the BTO particle is higher than that of the EP matrix. This phenomenon demonstrated that the negative charges are more likely to accumulate in the interface region. On the one hand, the interface is more electro-negative than the BTO particle. On the other hand, the interface traps introduced by adding BTO particles are easier to capture negative charges.

**Figure 8:**
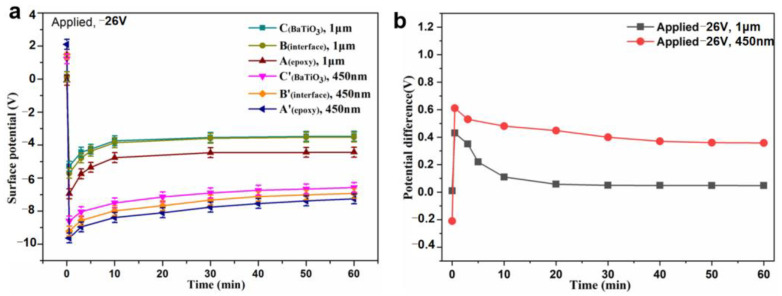
Variations in surface potential at different locations with time (**a**) and decay of potential difference between filler and interface with time (**b**).

The charge injection and dissipation behavior were analyzed at three typical locations of A (A’), B (B’), and C (C’), which, respectively, represent the matrix, interface, and filler (m-BTO or n-BTO), in order to clearly clarify the charge transport mechanism at the local region of BTO/EP composites under bias condition. Figure 8a showed the surface potential value decreases sharply at the three locations when the bias is applied and then increases slowly, finally becoming stable. The experiment results clearly manifested that the charged process around the BTO particle area is mainly done in the initial period of application of the external voltage. Figure 8b indicates that the potential difference between filler and interface initially increases rapidly and then decreases slowly, eventually approaching stable, which demonstrates that the electric field distribution around BTO particle is changed from one determined by permittivity to one determined by conductivity. The potential difference for different samples at t = 0 s is caused by the interface polarization due to the different permittivity between filler and matrix. It is worth noting that there is a slight drop in the potential difference value with time, indicating that the charge on the particle is neutralized by heteropolar carriers. Additionally, the stable state is basically achieved around the m-BTO particle at t = 20 min, while the n-BTO particle takes 40 min. This result illustrated that there is a discrepancy in conductivity around the m-BTO and n-BTO particles. 

The charge density *p*(*x*), electric field *E*(*x*), and charge amounts can be obtained from the surface potential data *φ*(*x*) by a KPFM test, and the calculating method has been in detail reported in the literature [49]. When the KPFM test was carried out for the solid dielectric, it was difficult to avoid the effect of noise data. In particular, the noise data were be amplified during the higher-order derivative’s calculation, resulting in erroneous results. Thus, a smoothing-derivative algorithm extended from the Savitzky–Golay algorithm combined with a numerical statistical method known as F-test [50] was developed to calculate the space charge distribution from the noisy potential data. Firstly, the appropriate polynomial order n and the length of a data window were determined for the polynomial function *f*(*x*) by Matlab software. Subsequently, the potential, electric field, and charge density were recalculated by the *f*(*x*) function [42,51,52]. This algorithm was described in the previous study [53].

(2) $$E\left(x\right)=-\frac{d\varphi \left(x\right)}{d\left(x\right)}$$

(3) $$\rho \left(x\right)=-\varepsilon_{0}\varepsilon_{r}\frac{d^{2}\varphi \left(x\right)}{dx^{2}}$$

(4) $$f\left(x\right)=a_{0}+a_{1}x+a_{2}x^{2}+a_{3}x^{3}+a_{4}x^{4}\cdots +a_{n}x^{n}$$

Here, $\varepsilon_{0}$ and $\varepsilon_{r}$ stands for vacuum permittivity and relative permittivity, respectively.

The local charge quantity *Q* of the material is described as:

(5) $$Q=\int_{ini}^{end}\rho \left(x\right)dx$$

where in, ini and end represent the initial data set and final data set, respectively. 

**Figure 9:**
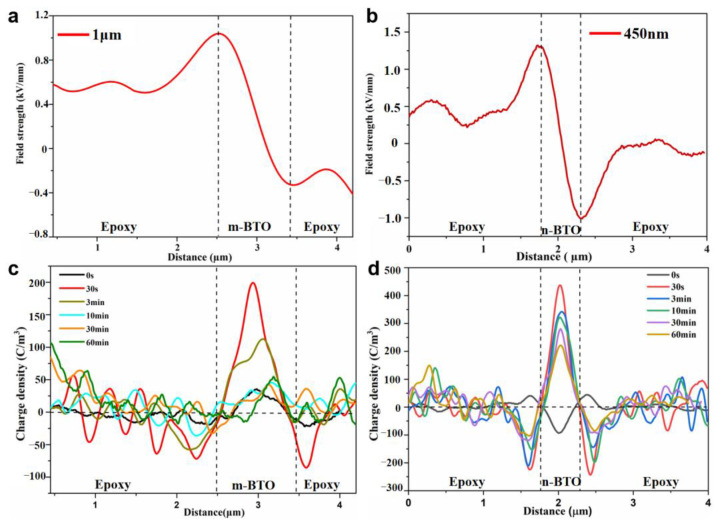
Field strength distribution around the m-BTO (**a**) and n-BTO (**b**) particle and the local charge density around m-BTO (**c**) and n-BTO (**d**) at different times under polarization.

Figure 9a,b manifested electric field strength distribution around the n-BTO and m-BTO particles. The electric field does not vary linearly due to that the charge gathered around the BTO particle affects the internal electric field in BTO/EP composites. The charge density profiles depicted that the BTO particle was positively charged, and negative charge gathered at the interfacial region, as shown in Figure 9c,d. Additionally, it is noticeable that the n-BTO particle has a relativity larger charge density compared with m-BTO particles. The reason for this phenomenon is that n-BTO and m-BTO particles have different dielectric constants. The conclusion also makes clear why the above result is that n-BTO/EP material has a larger breakdown field strength than that of m-BTO/EP material in DC breakdown experiments. We can also observe that the charge amount Q, which is approximately estimated, increases rapidly when the voltage is applied and then slightly decreases, finally approaching stable, as shown in Figure S6. The average decay rates of charge amount calculated at the m-BTO particle and interface region are about 0.725 × 10^−7^ C·m^−2^·s^−1^ and 0.369 × 10^−7^ C·m^−2^·s^−1^, respectively, which is slightly larger than that of n-BTO filler and interface (0.665 × 10^−7^ C·m^−2^·s^−1^ and 0.171 × 10^−7^ C·m^−2^·s^−1^, independently). Although the charge amount decreases slightly with time, the injected charge amount for n-BTO is always larger than that of m-BTO. The charge accumulation or dissipation rate *v* during the same time at the interface/filler is defined as

(6) $$\nu =\frac{Q_{1}/t_{1}+Q_{2}/t_{2}+Q_{3}/t_{3}+\cdot \cdot \cdot +Q_{n}/t_{n}}{n}$$

Here *Q_n_* is charge amount; *t_n_* is time; *n* is constant.

In addition, how the presence of BTO particles influences the surface potential distribution of epoxy composite was further stimulated by the finite element method (FEM) with COMSOL Multiphysics Software, as shown in Figure S7. It indicates that the charge accumulation around the filler is consistent with the results provided by KPFM analysis.

### 3.5. Charge Transport Behavior at the Interface Region of BTO/EP after Removal of Voltage

**Figure 10:**
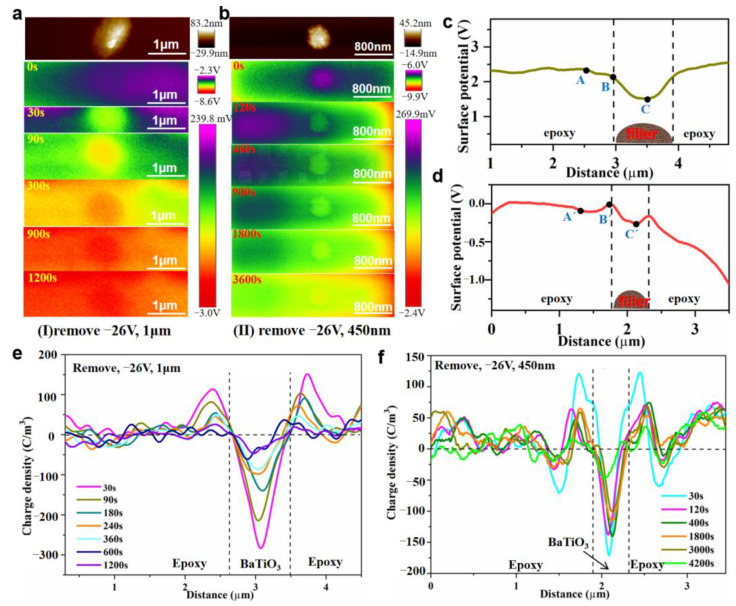
Local potential images of the m-BTO/EP (**a**) and the n-BTO/EP (**b**) after removing external voltage at various times; Surface potential distribution around the m-BTO particle (**c**) and n-BTO particle (**d**) after removal of the voltage; Local charge density curves of the m-BTO/EP (**e**) and the n-BTO/EP (**f**) in a short circuit.

The charge dissipation behavior around the single n-BTO and m-BTO particle were further investigated in BTO/EP composite materials in a short circuit. When the voltage was applied for 120 min, it was removed from the BTO/EP composites. Figure 10a,b intuitively reflects the local charge decay process at different times around the BTO filler. The potential images present a comparison of n-BTO and m-BTO particles. As time goes on, the interface of both m-BTO and EP, as well as n-BTO and EP, are not clearly visible in BTO/EP composites. Especially for the m-BTO/EP composite, the interface cannot be distinguished at t = 900 s, which revealed that the accumulated charge around the m-BTO particle dissipates quicker with respect to that of an n-BTO particle. In addition, the potential value of the filler is lower than that of the interface after the removal of voltage regardless of n-BTO or m-BTO particle, as shown in Figure 10c,d. Contrary to the charged case when the voltage was applied, in short-circuit conditions, negative charges accumulate on the BTO particle, and positive charges gather at the interface region, as illustrated in Figure 10e,f. Comprehensive analysis of the charge accumulation and dissipation behavior around BTO particles under polarization and depolarization process clearly showed that BTO, as a ferroelectric material, can undergo spontaneous polarization, so the charge accumulation around a BTO particle is mainly a comprehensive reflection of the interface polarization and spontaneous polarization. Under bias conditions, the particles are positively charged under the integrated polarization effect. After the removal of the voltage, there is a lag relationship between the spontaneous polarization and electric field, and due to the effect of remnant polarization, a negative net charge accumulates on the particles, which gradually dissipates with time.

**Figure 11:**
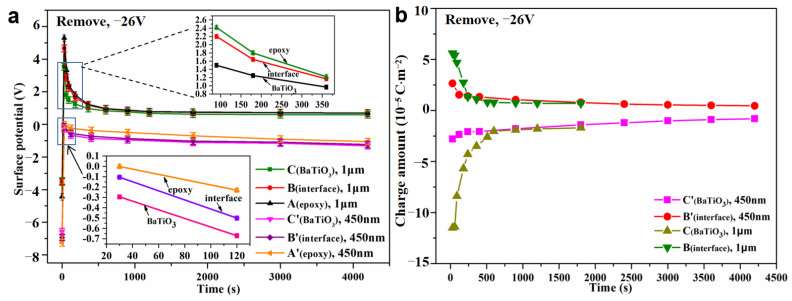
Variations of surface potential (**a**) and charge amount (**b**) at different locations with time after removal of the external voltage.

Figure 11a shows the evolution of surface potential at different locations with time in BTO/EP composites in a short circuit. The potential value for the BTO particle, interface, and matrix dropped abruptly within a very short time when the voltage was removed. There is no significant difference in the decay rate of potential at the three locations. What is more, the local charge amount initially decrease quickly and then reduce slightly, finally tend to be stable, as verified in Figure 11b. The reason for the above charge decay behavior is as follows: on the one hand, the traps with different energy levels can capture carriers in BTO/EP composites. Carriers captured usually stay for a very short time (10^−12^ s) in shallow traps and can migrate through hopping conduction [54]. However, the carriers caught in deep traps may stay for a few minutes to several days, even longer in epoxy composites [55]. On the other hand, the charge dissipation process is related to the depth of traps. The charge dissipation process is mainly completed by shallows traps in the initial period of removing voltage. As time goes on, the charge in shallow traps is almost dissipated completely, and then the deep traps play a dominant role in charge dissipation in BTO/EP composites. The calculated average charge dissipation rate at the m-BTO particle and interface are about 0.720 × 10^−6^ C·m^−2^·s^−1^ and 0.714 × 10^−6^ C·m^−2^·s^−1^, respectively, which is about one order of magnitude larger than that of an n-BTO particle (about 0.174 × 10^−7^ C·m^−2^·s^−1^ at filler and 0.294 × 10^−7^ C·m^−2^·s^−1^ at the interface). At the same time, it is worth noting that a typical electric double layer around the n-BTO particle can be found (Figure 10f) after the removal of the external voltage. The n-BTO particle has a strong binding effect on the charge at the interface, thus, leading to a slower charge dissipation rate around the particle.

**Figure 12:**
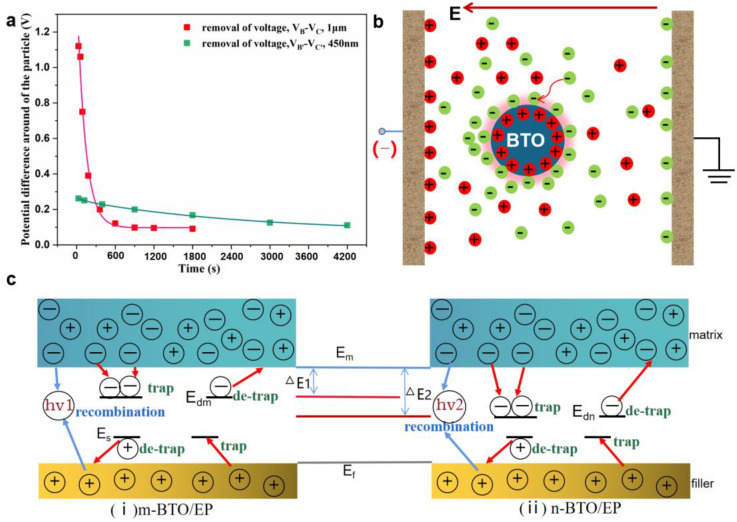
Potential difference between filler and interface around a single BTO particle in a short circuit (**a**), and schematic diagram of charge redistribution near a single BTO filler (**b**); Schematic diagram of a simplified model for charge migration in BTO/EP composites (**c**) (E_m_: the energy level of the matrix; E_dm_ and E_S_: the different energy level at interface region in n-BTO/EP composite; E_f_: the energy level of filler; E_dn_: the energy level at interface region in m-BTO/EP composite; ΔE1 and ΔE2 stand for the energy were required for the electrons de-trapping from the interface traps in m-BTO/EP and n-BTO/EP composite, separately).

In order to further analyze the interface trap characteristics for different BTO particles, the potential difference between the BTO particle and interface was also studied in a short circuit, as shown in Figure 12a. The m-BTO particle basically reaches a steady state after at t = 900 s, while the n-BTO particles still do not reach equilibrium at t = 4200 s, and at this time, the charge around the n-BTO particle is still dissipating. The absolute value of the potential difference changed by 0.16 V for an n-BTO particle at t = 4200 s. Concurrently, the potential difference changes by 1.10 V for an m-BTO particle at t = 1200 s. The decay rate of potential difference could reflect the interface trap depth in the dielectric composite. Therefore, the result reveals that the interface trap depth around the n-BTO is deeper than that of the m-BTO particle, which is consistent with the conjectured result by the PEA test.

The study of electrical properties at the interface region in dielectric composites is complex but interesting. Here the dynamic behavior of charge transfer was researched in BTO/EP composites, and the interface region around BTO particles is more capable of accumulation of negative charges when the negative polar voltage is applied, as shown in Figure 12b. The schematic diagram of a simplified model for the charge migration mechanism at the interface region is shown in Figure 12c. The addition of a filler introduces lots of traps with different energy levels (such as E_dn_, E_s_, E_dm_, et al.) into the BTO/EP composite. Either electrons or holes can be captured by traps, but the negative charge is more easily trapped by the interface traps. The negative charge captured at the interface region between n-BTO and matrix required more energy to de-trap from the traps than that of in m-BTO/EP composite material (ΔE2 > ΔE1). The positive or negative charge can be recombined with the heteropolar charge to reduce charge accumulation in the dielectric materials, improving the dielectric property of composites.

## 4. Conclusions

In this paper, the dielectric property of epoxy composites was improved by doping BTO particles. The breakdown field strength of BTO/EP composites displays a significant increase of approximately three times from ~100 kV/mm to ~300 kV/mm with 1.0 wt% BTO loading. The conductivity for BTO/EP composites also decreased, especially for n-BTO/EP composite, compared with pure EP. Concurrently, the interfacial charge transfer behavior in BTO/EP dielectric composites was elucidated by KPFM. The presence of BTO filler leads to a redistribution of charge. The electric field distribution around BTO particles is changed from one determined by permittivity to one determined by conductivity under bias conditions. The interface region is more capable of trapping negative charges under bias, and the charge injection and dissipation behavior exhibited significant discrepancies at different interfaces. The charge density of n-BTO particles is larger than that of m-BTO particles under the same bias conditions. The charge dissipation rate at the interface region in m-BTO/EP is faster than that of n-BTO/EP materials. In addition, n-BTO particles introduce deeper traps than that m-BTO in BTO/EP composites. The spontaneous polarization of BTO particles also has effects on charge distribution. Studies on the mechanism of interfacial charge transport at the micro level confirm the conjectures in PEA, conductivity, and breakdown field strength experiments. This paper clarified how the filler affects the charge transport in dielectric materials and provides experimental support for understanding the mechanism of interfacial interaction between filler and matrix in the dielectric material.

## Data Availability

The data presented in this study are available on request from the corresponding author.

## Supplementary Materials

The following supporting information can be downloaded at: https://www.mdpi.com/article/10.3390/nano13030406/s1, Figure S1: The Schematic representation of the test sample for KPFM measurement; Figure S2: (a) Height curve of electrode-Al and epoxy film. (b) Height diagram of Al-dielectric-Al; Figure S3:Diagram of KPFM experimental setup; Figure S4: Thermal properties analysis of samples: DSC (a), weight loss (b), and evolution of the interface region with particle size (c); Figure S5: (a) The permittivity and height of BaTiO_3_/EP composite in local region; (b) The XRD spectra of BaTiO3 particle; Figure S6: The change of charge amount at B/(B’) and C/(C’) with time under -26 V; Figure S7: Surface potential simulation by finite element method (FEM). (a) 3D image of m-BTO/EP and (c) n-BTO/EP. The simulated potential distribution of (b) m-BTO/EP and (d) n-BTO/EP.
