# Supplementary material for: Interfacial Insight of Charge Transport in BaTiO3/Epoxy Composites
Source: Nanomaterials (Basel). 2023 Jan 19;13(3):406. doi: 10.3390/nano13030406 (PMC9920443; doi:10.3390/nano13030406)
Supplement: Supplementary file 1 [file nanomaterials-13-00406-s001.zip › nanomaterials-2142405-supplementary.pdf]

Supporting Information

Interfacial Insight of Charge Transport in $BaTiO_3$/Epoxy Composites

Beibei Jia [1], Jun Zhou [1,*], Jiaxin Chen [1], Zixuan Zhang [1], Yang Wang [2], Zepeng Lv [1,*] and Kai Wu [1]

[1] Center of Nanomaterials for Renewable Energy, State Key Laboratory of Electrical Insulation and Power Equipment, Xi'an Jiaotong University, Xi'an 710049, China
[2] School of Electronics and Information, Xi'an Polytechnic University, Xi'an 710048, China
* Correspondence: zhoujun@mail.xjtu.edu.cn (J.Z.), lv.zepeng.insu@xjtu.edu.cn (Z.L.); Tel.: +86-029-8266-4480 (J.Z.)

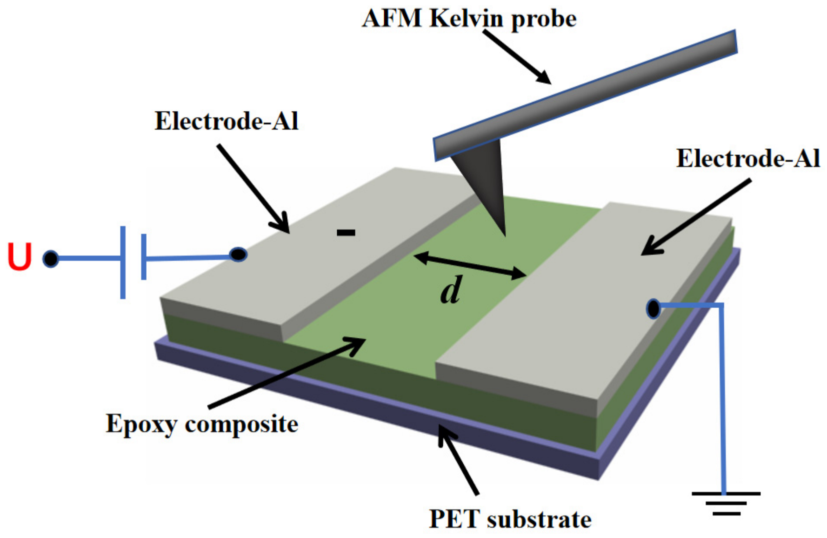

Figure S1. The Schematic representation of the test sample for KPFM measurement.

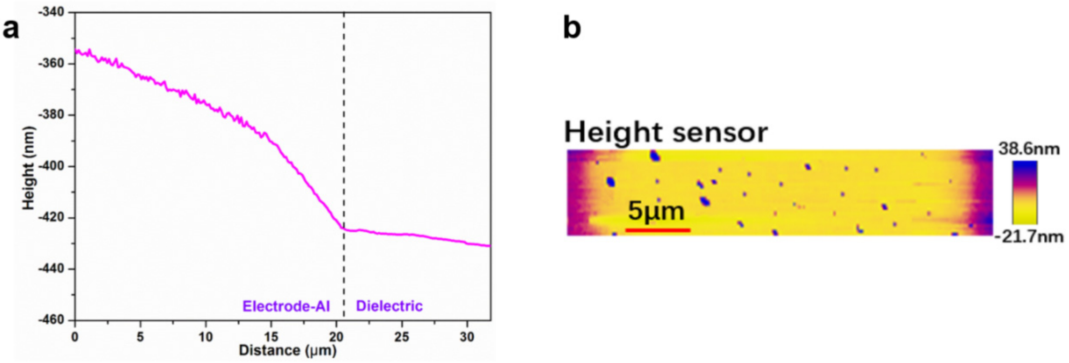

Figure S2. Height curve of electrode-Al and epoxy film(a); Height diagram of Al-dielectric-Al(b).

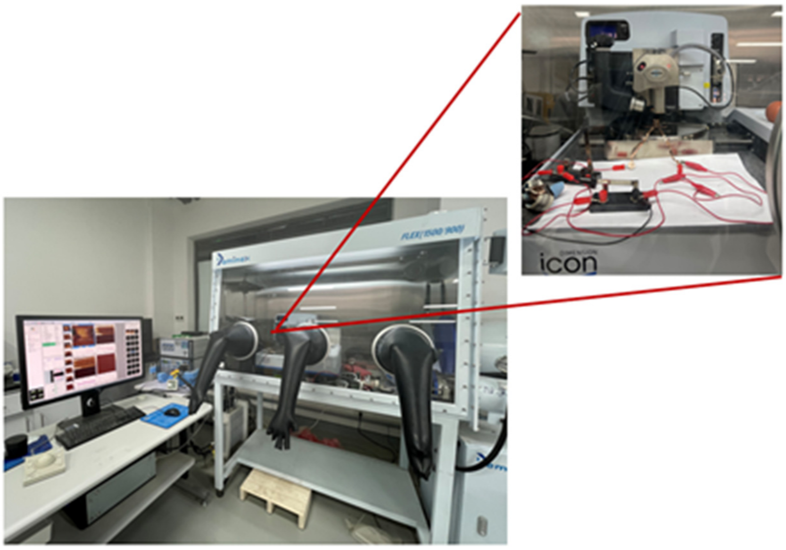

Figure S3. Diagram of KPFM experimental setup.

The heat flow curves measured by differential scanning calorimetry are demonstrated in **Figure S4a**. The results show that the endothermic peak temperature increased after the addition of the 1.0 wt% m-BTO filler in the temperature range of 25-180 °C, which represents the temperature of the main molecular chain movement lifts from 116.1 to 119.7 °C. It is ascribed to the reduction in the free volume at the interface caused by the winding of polymer chains. Moreover, the addition of n-BTO particles results in a reduction of glass transition temperature compared with pure epoxy, which is due to that as the BTO particle size decreases, the interface plays a dominant role, as shown in **Figure S4c**, the free volume of the interface region increases, promoting the movement of the molecular chain, so that the glass transition temperature of the polymer decreases.

The changes in weight loss for the different composites as a function of temperature under nitrogen are shown in **Figure S4b**. There are two main stages of weight loss. The first stage occurred at 188.6~305.3 °C, which represents the fracture of functional groups and the release of small molecules (CO/CO2/H2O, etc.). The second stage happened in the range of 305.3~524.5 °C, which was attributed to the fracture and degradation of skeleton of the macromolecule chain. These results could be suggested that the doping of n-BTO filler improves the stability of epoxy composites slightly.

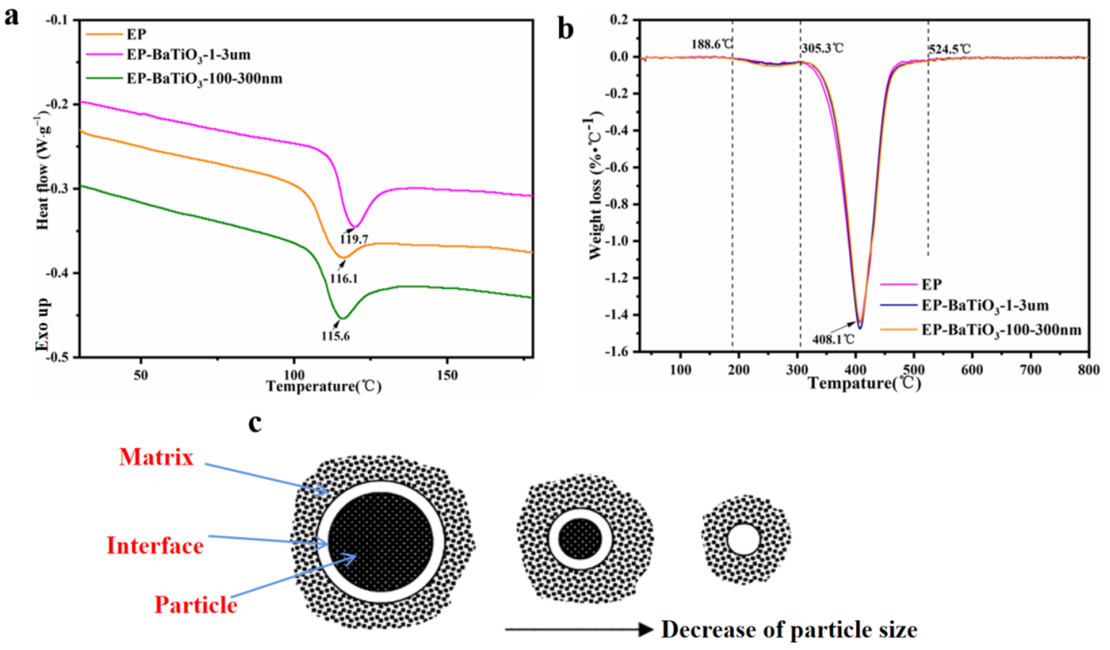

Figure S4. Thermal properties analysis of samples: DSC (a), weight loss (b), and evolution of the interface region with particle size(c).

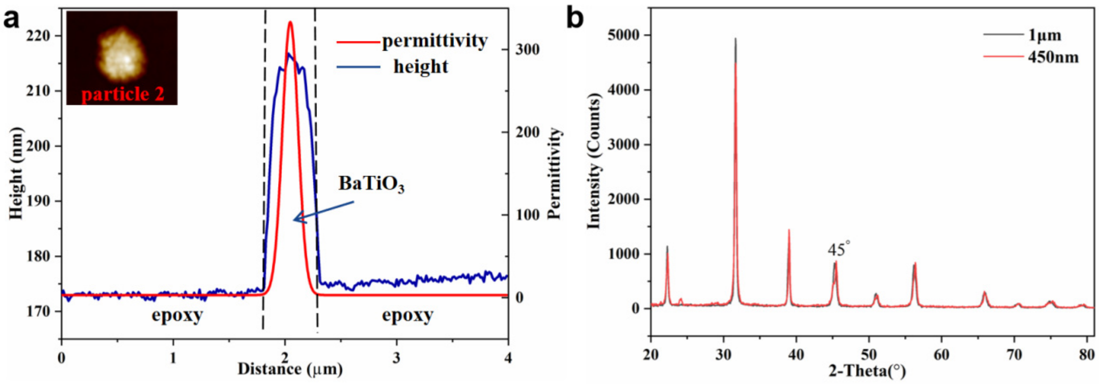

Figure S5. The permittivity and height of BaTiO3/EP composite in local region(a); The XRD spectra of BaTiO3 particle(b).

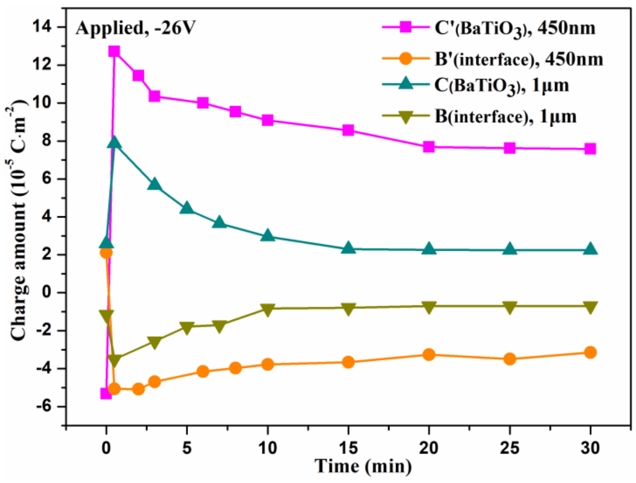

Figure S6. The change of charge amount at B/(B′) and C/(C′) with time under -26 V.

The charged state of around the particles under the electric field is verified from a simulation point of view, as shown in **Figure S7**. The local potential distribution around the filler is simulated with the "electrostatic" physical field module in COMSOL Multiphysics software. Firstly, the 3D geometric structure model is built. In the simulation experiment, the filler is approximately a sphere, and the matrix is regarded as hexahedral structure. Subsequently, input the radius of particle and its coordinate position in the matrix to form a combination of the two materials. And set the parameters of filler and epoxy matrix. At last, the dielectric constant and conductivity of the two materials are input to obtain 3D or 2D potential and electric field distribution. The simulation results also manifested that the potential of BTO particle is higher than that of matrix, that is, there is positive charge accumulation at the particle, and negative charge gathered at the interface region in BTO/EP composites. The charge accumulation characteristics are consistent with the results measured by KPFM method.

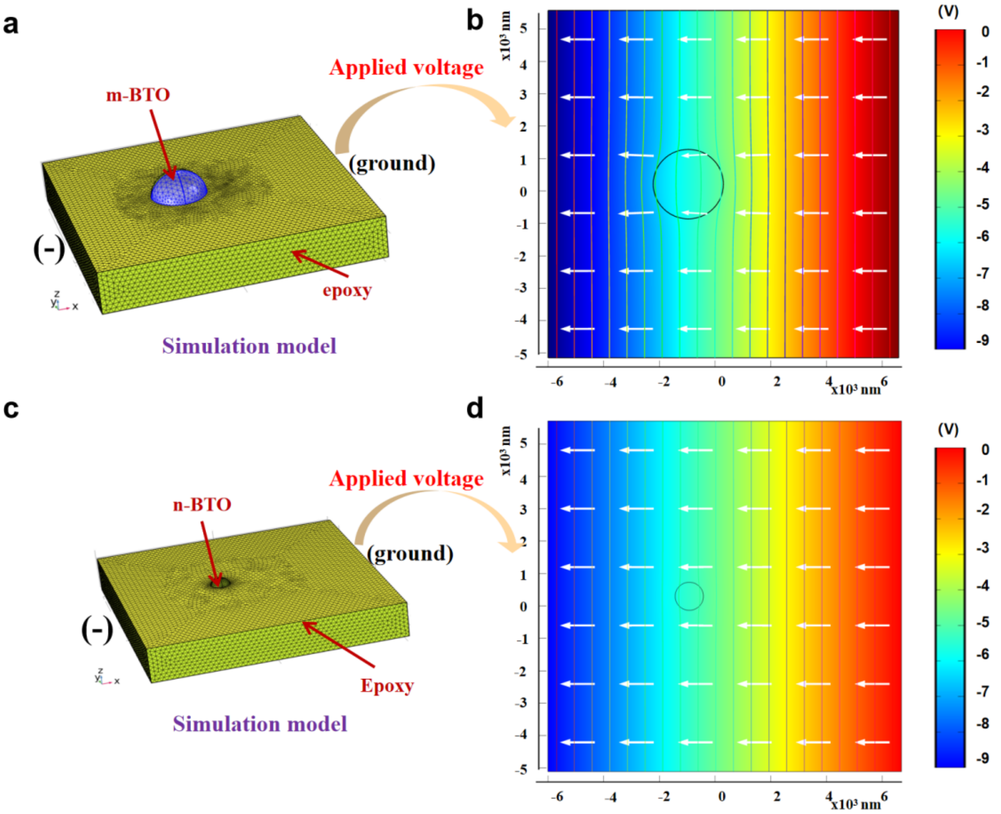

Figure S7. Surface potential simulation by finite element method (FEM). 3D image of m-BTO/EP(a), and n-BTO/EP(c); The simulated potential distribution of m-BTO/EP(b) and n-BTO/EP(d).
